# MRI morphological characteristics of lymph nodes in anal squamous cell carcinoma

**DOI:** 10.1007/s00261-023-04182-8

**Published:** 2024-02-06

**Authors:** Stephanie Gulevski, Anna Frennered, Elin Trägårdh, Martin P. Nilsson, Anders Johnsson, Pamela Buchwald, Lennart Blomqvist, Sophia Zackrisson

**Affiliations:** 1 Department of Clinical Sciences Malmö, Lund University, Malmö, Sweden; 2 Diagnostic Radiology, Department of Translational Medicine, Skåne University Hospital, Lund University, Malmö, Sweden; 3 Clinical Physiology and Nuclear Medicine, Department of Translational Medicine, Skåne University Hospital and Lund University, Malmö, Sweden; 4 Department of Haematology, Oncology and Radiation Physics, Skåne University Hospital, Lund, Sweden; 5 Department of Surgery, Skåne University Hospital, Malmö, Sweden; 6 Department of Radiation Physics/Nuclear Medicine, Karolinska University Hospital, Stockholm, Sweden; 7 Department of Molecular Medicine and Surgery, Karolinska Institute, Stockholm, Sweden

**Keywords:** Anus neoplasms, Lymphatic metastasis, Magnetic resonance imaging, Positron-emission tomography computed tomography

## Abstract

**Objectives:**

Pre-treatment staging of anal squamous cell carcinoma (ASCC) includes pelvic MRI and [18F]-fluorodeoxyglucose positron emission tomography with computed tomography (PET-CT). MRI criteria to define lymph node metastases (LNMs) in ASCC are currently lacking. The aim of this study was to describe the morphological characteristics of lymph nodes (LNs) on MRI in ASCC patients with PET-CT-positive LNs.

**Methods:**

ASCC patients treated at Skåne University Hospital between 2009 and 2017 were eligible for inclusion if at least one positive LN according to PET-CT and a pre-treatment MRI were present. All PET-CT-positive LNs and PET-CT-negative LNs were retrospectively identified on baseline MRI. Each LN was independently classified according to pre-determined morphological characteristics by two radiologists blinded to clinical patient information.

**Results:**

Sixty-seven ASCC patients were included, with a total of 181 PET-CT-positive LNs identified on baseline MRI with a median short-axis diameter of 9.0 mm (range 7.5–12 mm). MRI morphological characteristics of PET-CT-positive LNs included regular contour (87%), round shape (89%), and homogeneous signal intensity on T2-weighed images (67%). An additional 78 PET-CT-negative LNs were identified on MRI. These 78 LNs had a median size of 6.8 mm (range 5.5–8.0 mm). The majority of PET-CT-negative LNs had a regular contour, round shape, and a homogeneous signal that was congruent to the primary tumor.

**Conclusions:**

There are MRI-specific morphological characteristics for pelvic LNs in ASCC. PET-CT-positive and negative LNs share similar morphological features apart from size, with PET-CT-positive LNs being significantly larger. Further studies are needed to determine discrimination criteria for LNM in ASCC.

**Supplementary Information:**

The online version contains supplementary material available at 10.1007/s00261-023-04182-8.

## Introduction

Anal squamous cell carcinoma (ASCC) is relatively rare with an incidence of 2 per 100 000 individuals; however, the incidence is steadily rising in Western countries including Scandinavia and the US [1]. The 5-year survival rate is 60–80% and tumor stage according to TNM is the most important prognostic factor [2–5]. The presence of lymph node metastases (LNM) is reported clinically in about 30–50% of patients at diagnosis, while distant extrapelvic metastases are rare, reported in < 10% of patients at diagnosis [6–8].

Pelvic magnetic resonance imaging (MRI) is used for local staging and 18F-fluorodeoxyglucose (18F-FDG) positron emission tomography combined with computed tomography (PET-CT) is used for metastatic staging in ASCC. For regional lymph nodes (LNs), both MRI and PET-CT could be used, but no validated imaging criteria exist for LN evaluation in ASCC [9]. Nodal enlargement on cross-sectional imaging with a short-axis diameter of > 10 mm for mesorectal and > 15 mm for extramesorectal LNs, respectively, has been suggested as indicating LNM. However, the scientific evidence for these criteria is very limited. Furthermore, the presence of necrosis resulting in an inhomogeneous nodal enhancement on CT and MRI and signal intensity similar to the primary anal cancer tumor have been observed and suggested indicators of LNM [2, 10–12]. Additionally, morphologic nodal features as well as LN localization and hypermetabolism have been considered indicative of LNM [2, 13].

Concurrent chemoradiotherapy (CRT) is the standard treatment of ASCC. Histopathological confirmation of suspected LNM is thereby not achieved before treatment and responsiveness to CRT is measured by repeated imaging. TN stage and tumor location decide radiation dose and field including the primary tumor, suspicious LNM, and adjuvant dosage to adjacent LN locations. A total dose of 58 Gy is given to LNM exceeding 2 cm in diameter (50 Gy for nodes up to 2 cm), and 40 Gy to elective nodal stations including presacral, perirectal, internal iliac, and obturator nodes. Hence, correct nodal assessment is of relevance to ensure appropriate radiation therapy, minimizing the risk of over- and undertreatment [2, 14]. Nodal migration, a phenomenon marked by increased LN positivity detection over time with stable T-stage proportions between LN positive and negative patients, was observed in a meta-regression from 2017 [8]. Advanced imaging may contribute to nodal migration, potentially leading to N-stage misclassification. In ASCC, there is a discrepancy in LNM detection on PET-CT versus MRI [15–17]. This emphasizes the need for a deeper understanding of the morphological characteristics of LNs in ASCC patients treated primarily non-surgically, especially in relation to metabolic activity on PET-CT. We hypothesized that combined information from MRI and PET-CT could further improve nodal staging and support decision-making.

The aim of this study was to describe MRI morphological characteristics of PET-CT-positive and negative regional LNs in ASCC patients.

## Material and methods

### Study design, patient population, and lymph nodes

Patients were identified from a previously described study population, including all consecutive patients diagnosed with ASCC and treated with radiotherapy between August 1st, 2009 and December 31st, 2017 at Skåne University Hospital, Sweden. Ethical approval was obtained from the Swedish Ethical Review Authority (Dnr 2013/742 and 2019/02669) [18, 19]. Inclusion criteria were ASCC patients who underwent both PET-CT and pelvic MRI before radiotherapy, with at least one positive LN on baseline PET-CT. PET-CT-positive LNs were defined as LNs with a Deauville score (DS) of ≥ 3 (uptake of 18F-FDG greater than the uptake of the mediastinal blood pool), regardless of size. The time interval between baseline PET-CT and MRI scans was recorded, but no specific time limit was required for inclusion. Cases with poor image visualization due to low spatial resolution or artifacts were excluded. The absence of T2-weighed (T2W) imaging sequence on MRI or the presence of synchronous other benign or malignant pathology potentially influencing pelvic LNs led to exclusion. Furthermore, individual PET-CT-positive LNs were excluded from analysis if the nodal visualization was poor due small size (< 5 mm), or if deemed as tumor-related pathology rather than true LNs when assessed on MRI.

### MRI and PET-CT image acquisition

MRI scanners from different manufacturers were used (1.5-T scanners: Siemens Avanto, Symphony, Aera, Philips Intera and GE Optima and Signa; 3-T scanners: Siemens Trio, and GE Discovery). The routine MRI protocol for staging ASCC included standard T2W images in axial, sagittal, and two oblique directions. The slice thickness in axial T2W was in the range of 3–5 mm. When analyzing the LNs, the axial (non-oblique) T2W sequence was primarily used.

PET-CT acquisitions were performed using one of two different equipment: Discovery MI (GE Healthcare, Milwaukee, WI, USA), and Philips Gemini TF (Philips Healthcare, Cleveland, OH, USA). Fasting for at least 4 h before the examination and a glucose level ≤ 180 mg/dl was required. The accumulation time, defined as the duration between intravenous injection of 4 MBq/kg body weight of 18F-FDG and the initiation of image acquisition, was uniformly set at 60 min for all subjects. A PET scan was performed from the upper thighs to the base of the skull. In our clinical protocol, attenuation correction and anatomical correlation of PET images for radiotherapy treatment planning were conducted through either a diagnostic CT with intravenous and oral contrast or a low-dose CT without contrast. Integration with a low-dose CT was implemented when a diagnostic CT had not been performed within the preceding four weeks.

### Data collection and image analysis

Data on patient and tumor characteristics of patients with PET-CT-positive LNs had previously been collected from medical records, including age, sex, and clinical TNM status [19]. The baseline PET-CT scans were evaluated by a senior radiologist (AF), with complex cases subjected to additional review by a senior nuclear medicine physician (ET). All PET-CT-positive LNs were identified on pre-treatment MRI scans and independently assessed by two radiologists with 5 (AF) and 30 years (LB) of experience in evaluating pelvic MRI. The radiologists were blinded to clinical information other than the inclusion criteria, and all images were pseudonymized. A web-based zero foot-print Digital Imaging and Communications in Medicine viewer (Collective Minds Radiology (CMRAD), www.cmrad.com), was used for reviewing of imaging together with an interface. All findings on pre-treatment MRI scans corresponding to PET-CT-positive LNs previously described [15] were annotated numerically in CMRAD by AF. The radiologists evaluated the MRI findings separately. For each LN, MRI morphological features including size (short-axis axial plane diameter in mm), contour (regular or irregular), shape (round or oval (i.e., two-fold difference in transverse dimensions)), and fat content (present or not) were recorded according to a pre-determined protocol. The nodal signal was described as either inhomogeneous, homogeneous, or necrotic, and was compared to the signal of the primary tumor (same or not). Furthermore, any additional non-hypermetabolic (i.e., PET-CT-negative) LNs found on MRI by at least one radiologist were evaluated if large enough to be characterized (short-axis size of ≥ 5 mm used as threshold for evaluation) and analyzed separately. If the same PET-CT-negative LN was identified and assessed by both reviewers, the mean size value of the two radiologists (AF and LB) and the categorical values of AF were used for descriptive analysis. After independent assessment of the MRI findings by the two radiologists, consensus was reached regarding each finding as either LNM or other tumor-related pathology including tumor deposit or extramural vascular invasion.

### Statistical analysis

Patient and nodal characteristics are presented as numbers and proportions in percent for categorical data, or median with interquartile range for continuous data. Independent Samples Mann–Whitney *U* Test was used for median LN size comparison, with a significance level of 0.05. Interobserver statistical analyses were performed for all PET-CT-positive LNs. To assess the degree of agreement between the two radiologists, Cohen’s kappa statistics were calculated for categorical variables. Agreement of continuous data was analyzed using a Bland Altman plot. Statistical analyses were performed using IBM SPSS statistics software, version 27.

## Results

### Study cohort

**Fig. 1:**
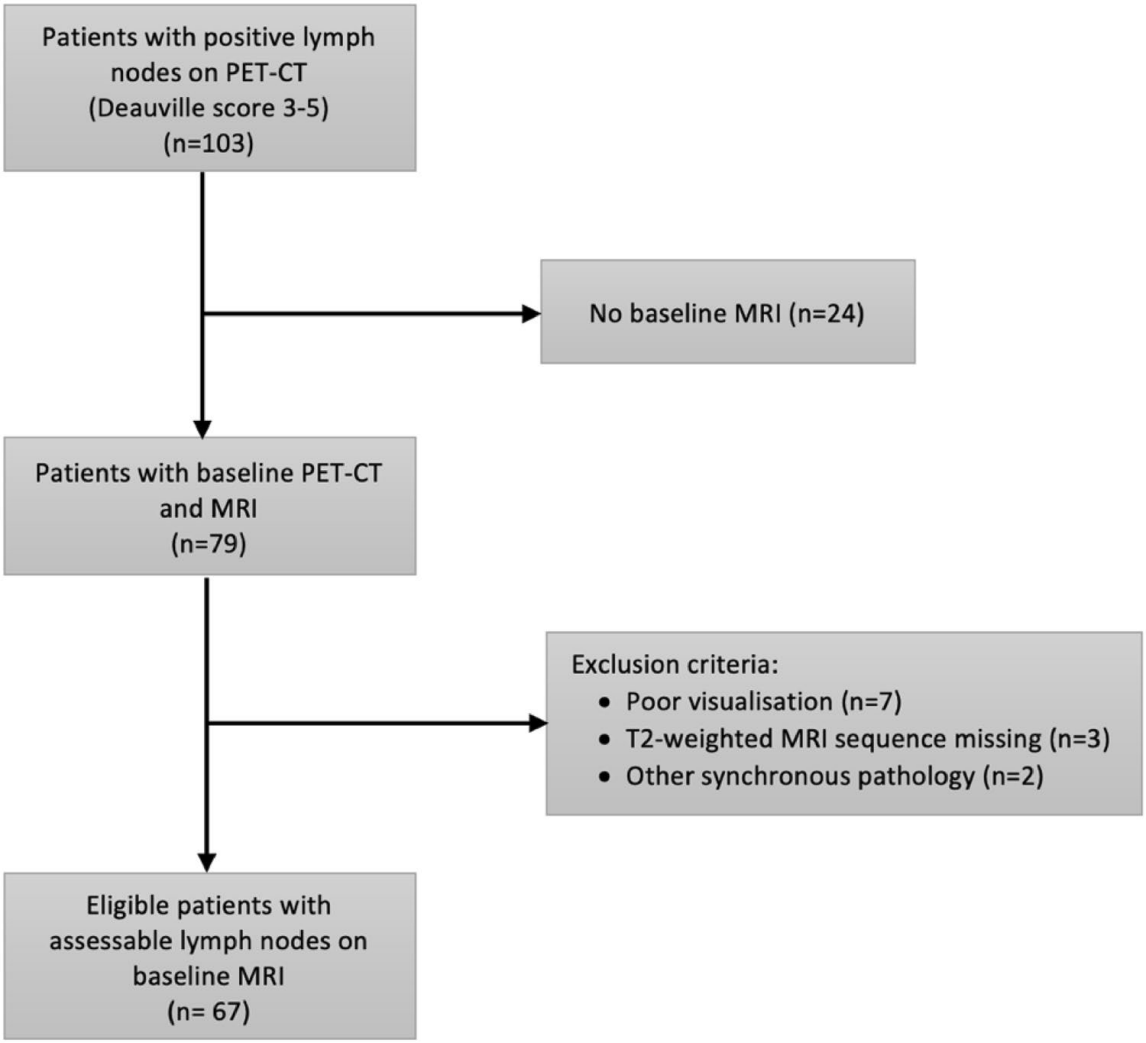
Flow chart of patient inclusion and exclusion

A total of 103 patients had PET-CT-positive LNs, of which 79 had a baseline MRI. Two patients were excluded because of synchronous chronic lymphocytic leukemia and perianal abscess, respectively, three patients due to incomplete MR-imaging acquisition protocol, and seven patients due to poor image visualization. Sixty-seven patients were ultimately included for analysis (Fig. 1). Patient and tumor characteristics of included and excluded patients are presented in Table 1.

**Table 1:** Patient and tumor characteristics

Characteristics	Included patients with PET-CT-positive lymph nodes (*n* = 67), *n* (%)	Excluded patients with PET-CT-positive lymph nodes (*n* = 36), *n* (%)
Age at diagnosis (years)	64 (57–70)	65 (56–73)
Gender (Ratio Female: Male)	3:1	6:1
BMI	25.9 (22.6–27.8)	24.9 (22.5–28.2)
HIV positive	1 (1)	0 (0)
Tumor localization		
Anal canal	6 (9)	4 (11.1)
Anal canal + rectum	24 (35.8)	7 (19.4)
Anal canal with perineal component	19 (28.4)	11 (30.6)
Anal canal with perianal component + rectum	17 (25.4)	11 (30.6)
Perianal	1 (1.5)	2 (5.6)
Rectum	0 (0)	1 (2.8)
Clinical T stage		
cT1	2 (3.0)	1 (2.8)
cT2	24 (35.8)	14 (38.9)
cT3	22 (32.8)	8 (22.2)
cT4	19 (28.4)	13 (36.1)
Clinical N stage		
cN0	5 (7.5)	6 (16.7)
cN1a	45 (67.2)	22 (61.1)
cN1b	0 (0)	2 (5.6)
cN1c	17 (25.4)	6 (16.7)
Clinical M stage		
cM0	57 (85.1)	32 (88.9)
cM1	10 (14.9)	4 (11.1)
Stage		
2a	3 (4.5)	3 (8.3)
2b	1 (1.5)	1 (2.8)
3a	20 (29.9)	11 (30.6)
3b	1 (1.5)	2 (5.6)
3c	32 (47.8)	15 (41.7)
4	10 (14.9)	4 (11.1)
Time between biopsy-verified diagnosis and MRI scan (days)*	13 (5–17)	
Time between MRI and PET-CT (days)*	19 (8–27)	

Categorical data are presented as number and percentage; continuous data are presented as median and interquartile range. TNM stage is presented according to TNM classification 8th edition, as judged by treating clinicians at time of diagnosis

BMI: body mass index, HIV: human immunodeficiency virus

*Not presented for excluded patients

### Characteristics of PET-CT-positive lymph nodes on MRI

**Fig. 2:**
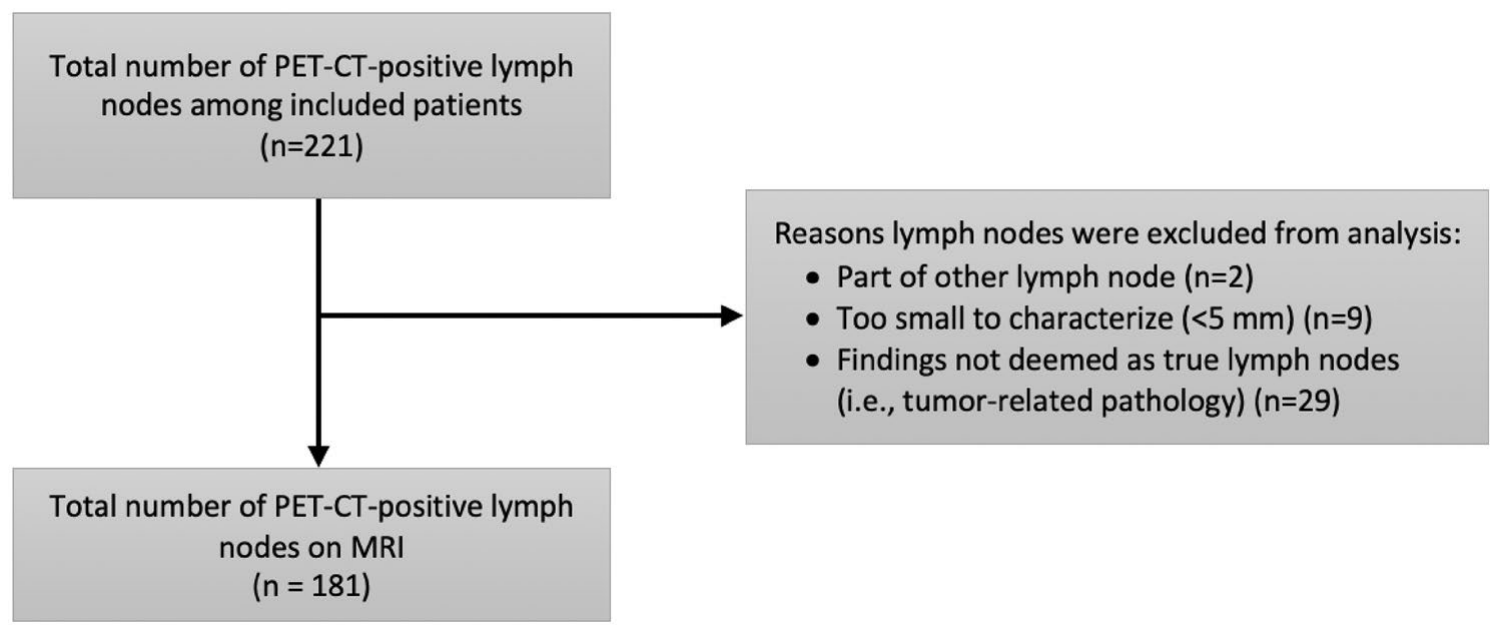
Flow chart of lymph node inclusion and assessment

**Fig. 3:**
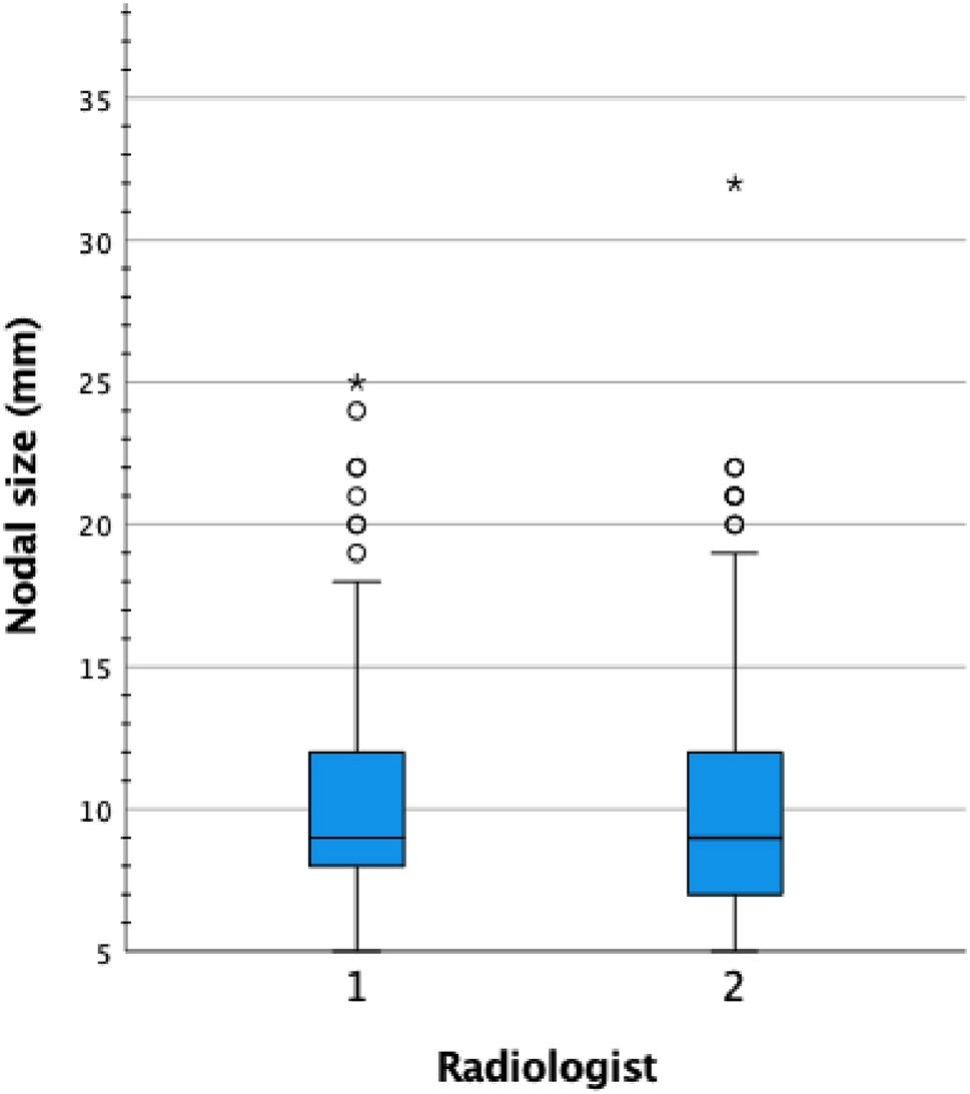
Size distribution of PET-CT-positive lymph nodes on MRI

A total of 181 LNs were analyzed after exclusion of non-assessable LNs (Fig. 2). The median size of PET-CT-positive LNs was 9.0 mm (7.5–12 mm). The size distribution of PET-CT-positive LNs on MRI is presented in Fig. 3.

**Fig. 4:**
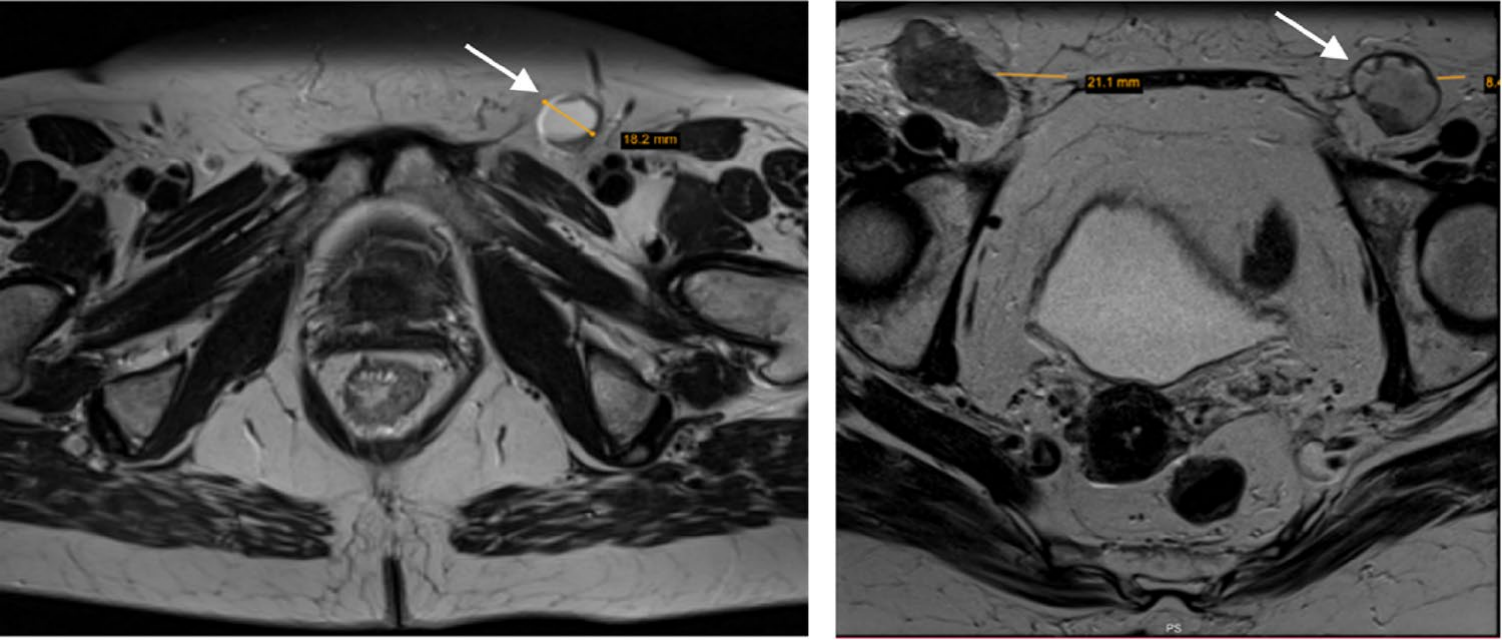
Two examples of PET-CT-positive left inguinal lymph nodes with large central necrosis on T2-weighted MR-image (white arrows)

The distribution of categorical morphological nodal characteristics of PET-CT-positive LNs is summarized in Table 2. On MRI, most PET-CT-positive LNs had a nodal signal intensity that was visually the same as for the primary tumor (76%), a round shape (89%), a regular contour (87%), and a homogeneous nodal signal (67%). Necrotic content was noted in 11% of all PET-CT-positive LNs, most commonly in inguinal LNs as exemplified in Fig. 4. The regional anatomical distribution of PET-positive lymph nodes is presented in Table A2, Appendix.

**Table 2:** Distribution of morphological characteristics of PET-CT-positive lymph nodes on MRI

Characteristics	PET-CT-positive lymph nodes on MRI (*n* = 181)			
	*Radiologist 1* (AF)	*Kappa* (95% CI)	*Radiologist 2* (LB)	*Mean value of radiologist 1* + *2*
Size				
Median (mm)	9.0 (8.0–12.0)		9.00 (7.0–12.0)	9.0 (7.5–12.0)
Nodal signal compared to primary tumor		0.48 (0.33–0.62)		
Same	145 (80.1)		129 (71.3)	(75.7)
Not the same	33 (18.2)		49 (27.1)	(22.8)
Primary tumor not visible on MRI	3 (1.7)		3 (1.7)	(1.7)
Contour		0.13 (0.00–0.26)		
Regular	176 (97.2)		140 (77.3)	(87.3)
Irregular	5 (2.8)		41 (22.7)	(12.7)
Nodal signal		0.39 (0.28–0.51)		
Inhomogeneous	34 (18.8)		45 (24.9)	(21.8)
Homogeneous	127 (70.2)		116 (64.1)	(67.2)
Necrotic	20 (11.0)		20 (11.0)	(11.0)
Shape		0.33 (0.20–0.53)		
Oval	20 (11.0)		20 (11.0)	(11.0)
Round	161 (89.0)		161 (89.0)	(89.0)
Fat content		0.25 (0.03–0.54)		
Present	13 (7.2)		2 (1.1)	(4.1)
Absent	168 (92.8)		179 (98.9)	(95.9)

Categorical data are presented as number and percentage; continuous data are presented as median and interquartile range. Cohen’s Kappa coefficients are presented for categorical variables

MRI morphological characteristics of PET-CT-positive LNs categorized in size categories of 5–8 mm and ≥ 9 mm, respectively, are presented in Table A1, Appendix.

### Characteristics of PET-CT-negative lymph nodes on MRI

In addition to the 181 PET-CT-positive LNs, 78 PET-CT-negative LNs ≥ 5 mm were identified on MRI by at least one of the reviewers. The morphological characteristics of these nodes are summarized in Table 3. The median size of PET-CT-negative LNs (6.8 mm) was significantly lower compared to the median size of PET-CT-positive LNs (*p* < 0.001). Most PET-CT-negative LNs had a homogeneous signal intensity (80%) and had the same signal intensity as the primary tumor (67%). The most predominant characteristics for these nodes were round shape (97%) with a regular contour (93%). Only one out of 78 PET-CT-negative LNs was deemed necrotic.

**Table 3:** Nodal characteristics of additional findings of PET-CT-negative lymph nodes on MRI

Characteristics	PET-CT-negative lymph nodes on MRI
Total number of lymph nodes	*n* = 78
Size	
Median (mm)	6.75 (5.5–8.0)
Nodal signal compared to primary tumor	
Same	52 (66.7)
Not the same	24 (30.8)
Primary tumor not visible on MRI	2 (2.6)
Contour	
Regular	72 (92.6)
Irregular	6 (7.7)
Nodal signal	
Inhomogeneous	15 (19.2)
Homogeneous	62 (79.5)
Necrotic	1 (1.2)
Shape	
Oval	2 (2.6)
Round	76 (97.4)
Fat content	
Present	2 (2.6)
Absent	76 (97.4)

Categorical data are presented as number and percentage; continuous data are presented as median and interquartile range

### Interobserver agreement

**Fig. 5:**
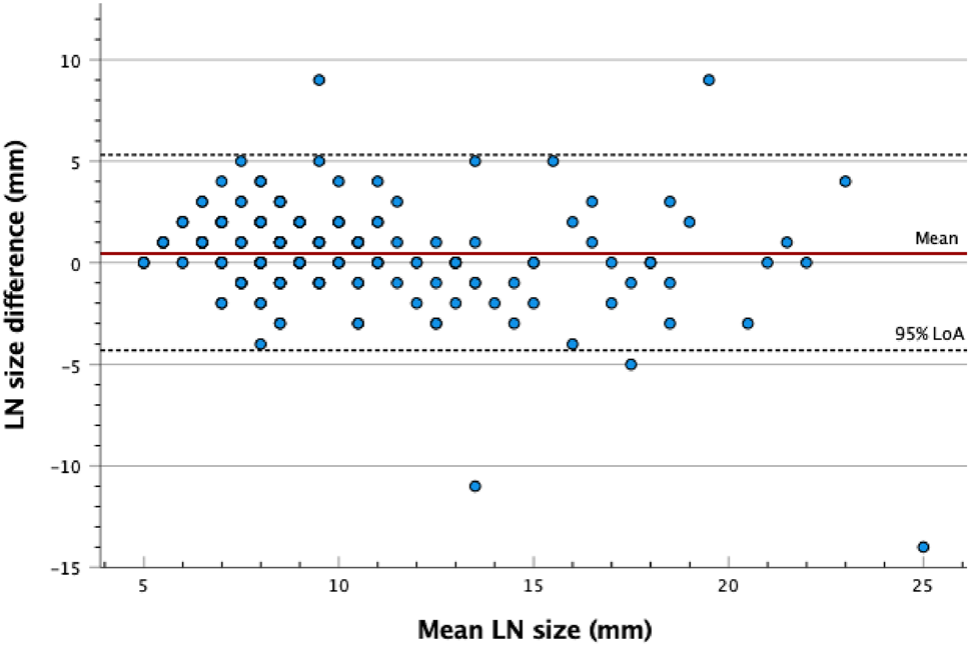
Bland–Altman plot of nodal size agreement between the two radiologists showing how the differences in lymph node (LN) size assessment correlate to nodal size. The red line represents the mean of the differences (0.5 mm) between the two radiologists. The dashed lines show the upper and lower 95% limits of agreement (LoA)

Interobserver agreement between the two radiologists was moderate for nodal signal compared to primary tumor (kappa 0.48), and poor regarding the rest of the morphological characteristics analyzed (Table 2). Bland–Altman statistics were performed to calculate systematic bias in nodal size evaluation, with a mean difference in nodal size between reviewers of 0.5 mm (limits of agreement – 4.3 to 5.3), presented in Fig. 5.

## Discussion

This study evaluated MRI morphology of LNs in ASCC patients with at least one PET-CT positive LN before treatment and found that both PET-CT-positive and negative LNs were predominantly characterized as homogeneous with nodal signal similar to the primary tumor. Most LNs, both PET-CT-positive and negative, showed round and regular morphology. As previous research on nodal disease in ASCC is scarce, these observations are novel and provide a foundation for future research on imaging criteria.

A substantial amount of PET-CT-negative LNs was additionally found on MRI (*n* = 78), which might indicate that not all LNMs in ASCC can be identified with PET-CT alone. The median size of PET-CT-negative LNs on MRI was statistically smaller than for PET-CT-positive nodes, despite sharing similar morphological characteristics. The smaller sizes of PET-CT-negative LNs could, at least partially, explain why metabolic uptake above the cut-off (DS ≥ 3) cannot be detected, indicating a potential threshold of size for LN detection on PET-CT. Hence, neither size nor hypermetabolism alone (defined as DS ≥ 3) seems to be reliable predictors of LNM in ASCC. According to the European Society of Gastrointestinal and Abdominal Radiology (ESGAR) consensus guidelines for nodal staging in rectal cancer, criteria for malignant nodes include LNs with a short-axis diameter of 5–8 mm and at least two suspicious morphological characteristics [20]. We therefore separated all PET-CT-positive LNs in size categories of 5–8 mm and ≥ 9 mm, respectively. The data presented in Table A1, Appendix, demonstrate that smaller PET-CT-positive LNs measuring 5–8 mm (*n* = 66) exhibit MRI morphological characteristics suggestive of pathology. As PET-CT could potentially miss smaller pathological LNs with insufficient 18F-FDG-uptake, multimodal imaging is of importance when assessing LNs in ASCC, where suspicious MRI morphological criteria could be applied for smaller LNs 5–8 mm similarly to the ESGAR guidelines for rectal cancer.

To our knowledge, this is the first study describing MRI morphological characteristics independent of PET metabolism in the setting of ASCC. The patient cohort was large considering the rarity of the disease and consisted of patients treated consecutively at a single institution with routine use of PET-CT. Nodal staging guidelines for primary rectal cancer may have been applied for nodal evaluation in ASCC, which in our opinion should not be done, considering the different anatomical and histological origins of rectal adenocarcinomas compared to ASCC. Morphological criteria such as round shape, irregular contour, loss of fatty hilum, and mixed/heterogeneous nodal signal intensity have all been proposed for detection of LNMs in rectal cancer before treatment [20–22]. In contrast to previous morphological findings for LNM in rectal cancer, the majority of all PET-CT-positive LNs in our ASCC patient cohort were judged to have a homogeneous nodal signal and a regular border, with similar characteristics seen in PET-CT-negative LNs identified on MRI. These results support that LNMs in the context of ASCC differ at least partly from LNMs seen in rectal adenocarcinomas, why the same criteria cannot be applied.

The study methodology poses some limitations. Firstly, and most importantly, nodal evaluations were of subjective nature without the possibility to compare to a reference standard, potentially introducing observer bias. Histopathology commonly serves as the gold standard reference in other diseases where surgery is the primary treatment. However, in ASCC, this reference standard is not obtainable since CRT is the standard treatment given and primary surgery is rare. In our cohort, none of the LNs were biopsied before CRT and locoregional nodal recurrence was rare after CRT, with confirmed histopathological metastasis in only two out of four patients who underwent either biopsy or surgical resection. For these reasons, the actual presence of metastases within the LNs detected with PET-CT and MRI remains unknown. Characteristics of PET-CT-positive and PET-CT-negative LNs were therefore presented descriptively, without the use of statistical analyses for comparison of LNs. However, only patients with at least one PET-positive LN were selected for inclusion in this study, which might increase the likelihood that additional PET-CT-negative LNs found on MRI are actual LNM containing micrometastases not reaching the cut-off value for hypermetabolism on PET-CT due to smaller size.

Secondly, different generations of PET-CT cameras and MRI scanners were used throughout the study period and patients were excluded if a baseline MRI scan was missing or if the image visualization was poor, which may have introduced selection bias. For this reason, patient characteristics of excluded patients were presented, and no major differences were noted.

Additionally, interobserver agreement for categorical morphological characteristics was poor to moderate, which is in accordance with previous results of interobserver agreement of nodal evaluation on MRI in ASCC [23]. This uncertainty underscores the importance of future studies to identify reliable nodal features. Possible contributors to the interobserver variability include the subjectivity involved in visual evaluation of nodal characteristics and varying experience of the reviewers. However, the overall distribution of morphological characteristics among PET-CT-positive LNs was relatively similar between the two reviewers. For nodal size, the random variation in size difference was relatively constant around the mean nodal size difference, and neither of the reviewers seemed to systematically under- or overestimate nodal size.

Another study limitation was that the anatomical location and symmetrical distribution of LNs were not considered when presenting nodal characteristics, as the amount of LNs assessed was deemed too low to find significant morphological differences in various pelvic locations or asymmetry between lymph nodes on different sides. The current patient cohort was used in a previous study, showing that PET-CT-positive LNs were most prevalent in the inguinal followed by the perirectal areas [18]. It is possible that inguinal LNMs differ morphologically from mesorectal LNMs. Even if subgroup analysis on location was not performed, we did observe that inguinal LNs tended to be necrotic to a greater extent compared to perirectal nodes. Necrosis was almost exclusively seen in PET-CT-positive LNs and is seemingly an important feature of LNMs in ASCC.

Importantly, we described MRI morphological characteristics of PET-CT-positive and negative regional LNs in ASCC patients (Tables 2 and 3), but their value for radiation therapy planning remains untested and needs to be examined in prospective clinical trials.

## Conclusion

Most regional LNs in ASCC patients with PET-positive LNs have a homogeneous nodal signal comparable to the primary tumor, and are morphologically characterized by a round and regular shape on MRI, regardless of nodal metabolic activity on PET-CT. These findings differ from morphological MRI criteria used for LNM detection in rectal adenocarcinomas. The combined utilization of PET-CT and MRI is recommended for nodal assessment, especially as smaller nodes (5–8 mm) may lack FDG-avidity but still display morphological features suggestive of malignancy. Further validation of our suggested morphological observations of regional LNs in ASCC is warranted.

### Supplementary Information

Below is the link to the electronic supplementary material.

Supplementary file1 (DOCX 24 KB)

## Abbreviations

ASCC: Anal squamous cell carcinoma
CMRAD: Collective Minds Radiology
CRT: Chemoradiotherapy
LNM: Lymph node metastases
LNs: Lymph nodes
DS: Deauville score
T2W: T2-weighed

## References

[1] Islami F, Ferlay J, Lortet-Tieulent J, Bray F, Jemal A (2017). International trends in anal cancer incidence rates. International journal of epidemiology..

[2] Nationellt vårdprogram analcancer. Regionala Cancercentrum i samverkan. https://kunskapsbanken.cancercentrum.se/diagnoser/analcancer/vardprogram/

[3] Ajani JA, Winter KA, Gunderson LL, Pedersen J, Benson AB, Thomas CR (2009). US intergroup anal carcinoma trial: tumor diameter predicts for colostomy. J Clin Oncol..

[4] Ajani JA, Winter KA, Gunderson LL, Pedersen J, Benson AB, Thomas CR (2010). Prognostic factors derived from a prospective database dictate clinical biology of anal cancer: the intergroup trial (RTOG 98–11). Cancer..

[5] Glynne-Jones R, Sebag-Montefiore D, Adams R, Gollins S, Harrison M, Meadows HM (2013). Prognostic factors for recurrence and survival in anal cancer. Cancer..

[6] Glynne-Jones R, Nilsson PJ, Aschele C, Goh V, Peiffert D, Cervantes A (2014). Anal cancer: ESMO-ESSO-ESTRO clinical practice guidelines for diagnosis, treatment and follow-up. Radiother Oncol..

[7] Johnsson A, Norman D, Angenete E, Cavalli-Bjorkman N, Lagerback C, Leon O, et al. Anal cancer in Sweden 2015–2019. Implementation of guidelines, structural changes, national registry and early results. Acta Oncol. 2022;61(5):575–82. 10.1080/0284186X.2022.2048069 35274596

[8] Sekhar H, Zwahlen M, Trelle S, Malcomson L, Kochhar R, Saunders MP (2017). Nodal stage migration and prognosis in anal cancer: a systematic review, meta-regression, and simulation study. Lancet Oncol..

[9] Golia Pernicka JS, Rauch GM, Gangai N, Bates DDB, Ernst R, Hope TA (2023). Imaging of Anal Squamous Cell Carcinoma: Survey Results and Expert Opinion from the Rectal and Anal Cancer Disease-Focused Panel of the Society of Abdominal Radiology. Abdom Radiol (NY)..

[10] Ciombor KK, Ernst RD, Brown G (2017). Diagnosis and Diagnostic Imaging of Anal Canal Cancer. Surg Oncol Clin N Am..

[11] Golia Pernicka JS, Sheedy SP, Ernst RD, Minsky BD, Ganeshan D, Rauch GM (2019). MR staging of anal cancer: what the radiologist needs to know. Abdom Radiol (NY)..

[12] Roach SC, Hulse PA, Moulding FJ, Wilson R, Carrington BM (2005). Magnetic resonance imaging of anal cancer. Clin Radiol..

[13] Rao S, Guren MG, Khan K, Brown G, Renehan AG, Steigen SE (2021). Anal cancer: ESMO Clinical Practice Guidelines for diagnosis, treatment and follow-up(☆). Ann Oncol..

[14] Ng M, Leong T, Chander S, Chu J, Kneebone A, Carroll S (2012). Australasian Gastrointestinal Trials Group (AGITG) contouring atlas and planning guidelines for intensity-modulated radiotherapy in anal cancer. Int J Radiat Oncol Biol Phys..

[15] Di Carlo C, di Benedetto M, Vicenzi L, Costantini S, Cucciarelli F, Fenu F (2021). FDG-PET/CT in the Radiotherapy Treatment Planning of Locally Advanced Anal Cancer: A Monoinstitutional Experience. Front Oncol..

[16] Bhuva NJ, Glynne-Jones R, Sonoda L, Wong WL, Harrison MK. To PET or not to PET? That is the question. Staging in anal cancer. Annals of Oncology. 2012;23(8):2078–82. 10.1093/annonc/mdr599 22294527

[17] Manafi-Farid R, Kupferthaler A, Wundsam H, Gruber G, Vali R, Venhoda C (2020). Additional Value of 2-[18F] FDG PET/CT Comparing to MRI in Treatment Approach of Anal Cancer Patients. Journal of Clinical Medicine..

[18] Frennered A, Scherman J, Buchwald P, Johnsson A, Sartor H, Zackrisson S (2021). Patterns of pathologic lymph nodes in anal cancer: a PET-CT-based analysis with implications for radiotherapy treatment volumes. BMC Cancer..

[19] Nilsson MP, Nilsson ED, Johnsson A, Leon O, Gunnlaugsson A, Scherman J (2020). Patterns of recurrence in anal cancer: a detailed analysis. Radiation Oncology..

[20] Beets-Tan RGH, Lambregts DMJ, Maas M, Bipat S, Barbaro B, Curvo-Semedo L (2018). Magnetic resonance imaging for clinical management of rectal cancer: Updated recommendations from the 2016 European Society of Gastrointestinal and Abdominal Radiology (ESGAR) consensus meeting. Eur Radiol..

[21] Kim JH, Beets GL, Kim MJ, Kessels AG, Beets-Tan RG (2004). High-resolution MR imaging for nodal staging in rectal cancer: are there any criteria in addition to the size?. Eur J Radiol..

[22] Brown G, Richards CJ, Bourne MW, Newcombe RG, Radcliffe AG, Dallimore NS (2003). Morphologic predictors of lymph node status in rectal cancer with use of high-spatial-resolution MR imaging with histopathologic comparison. Radiology..

[23] H. Sekhar RK, B. Carrington, M. van Herk, T. Kaye, D. Tolan, D. Sebag-Montefiore, A. G. Renehan. Poster Abstracts. P063: Two vs five MR morphological criteria for nodal detection in patients with anal cancer. Colorectal Disease. 2018;20(S7):25.

