# Supplementary material for: MRI morphological characteristics of lymph nodes in anal squamous cell carcinoma
Source: Abdom Radiol (NY). 2024 Feb 6;49(4):1042–50. doi: 10.1007/s00261-023-04182-8 (PMC10954875; doi:10.1007/s00261-023-04182-8)
Supplement: Supplementary file 1 — Supplementary file1 (DOCX 24 KB) [file 261_2023_4182_MOESM1_ESM.docx]

**Appendix**

**Table A1.** MRI morphological characteristics of PET-CT-positive lymph nodes (n=181), according to size categories of 5-8 mm and ≥ 9 mm. Numbers are presented as proportion (%) of lymph nodes in each size category.

**Reviewer 1.**

| Nodal size | Nodal signal same as the primary tumor | Homogenous signal intensity | Regular contour | Round shape |
| --- | --- | --- | --- | --- |
| 5-8 mm (n=66) | 77.3% | 69.7% | 81.8% | 98.5% |
| ≥ 9 mm (n=115) | 81.7% | 70.4% | 93.0% | 96.5% |

**Reviewer 2.**

| Nodal size | Nodal signal same as the primary tumor | Homogenous signal intensity | Regular contour | Round shape |
| --- | --- | --- | --- | --- |
| 5-8 mm (n=66) | 75.8% | 65.2% | 81.8% | 81.8% |
| ≥ 9 mm (n=115) | 68.7% | 63.5% | 93.0% | 74.8% |

**Table A2.** Regional distribution of PET-positive lymph nodes (n=181). Numbers are presented as proportion (%) of PET-positive lymph nodes in each anatomical region.

| Nodal region | n (%) |
| --- | --- |
| Inguinal | 102 (56.4) |
| Perirectal | 21 (11.6) |
| Internal iliac | 22 (12.2) |
| External iliac | 26 (14.4) |
| Common iliac | 5 (2.8) |
| Para-aortic | 5 (2.8) |

**Figure A1**. PET-CT negative lymph nodes identified on MRI by reviewer 1 and reviewer 2.

78 PET-negative lymph nodes identified on MRI

52 identified by both readers

3 identified by R1 only

23 identified by R2 only
